# Predictive Value of Heart-Type Fatty Acid-Binding Protein for Mortality Risk in Critically Ill Patients

**DOI:** 10.1155/2022/1720414

**Published:** 2022-12-27

**Authors:** Ye Zhang, Jia Wang, Bao-Zhong Yu, Mao-Lin Chen, Yu-Dan Cao, Bing Wei, Jun-Yu Wang

**Affiliations:** Emergency Medicine Clinical Research Center, Beijing Chaoyang Hospital, Capital Medical University, & Beijing Key Laboratory of Cardiopulmonary Cerebral Resuscitation, Clinical Center for Medicine in Acute Infection, Capital Medical University, China

## Abstract

**Objective:**

Our study assessed the predictive value of heart-type fatty acid-binding protein (H-FABP) for critically ill patients.

**Methods:**

150 critically ill patients admitted to the emergency department of Beijing Chaoyang Hospital, Capital Medical University, were included in our study from August 2021 to April 2022. Serum H-FABP, procalcitonin (PCT), lactate (LAC), and other markers were determined within 1 h after admission. The Sequential Organ Failure Assessment (SOFA) score and the Acute Physiology and Chronic Health Evaluation II (APACHE II) were calculated. The independent predictors of 28-day mortality in critically ill patients were analyzed by logistic regression, and the receiver operating characteristic curve (ROC) was used to analyze the predictive value for 28-day mortality in critically ill patients.

**Results:**

Age, APACHE II, SOFA, GCS, LAC, H-FABP, IL-6, Scr, and D-dimer were significantly different in the nonsurvivor vs. survivor groups (*P* < 0.05), with H-FABP correlating with cTNI, Scr, PCT, and SOFA scores (*P* < 0.05). Logistic regression analysis showed that H-FABP, APACHE II, LAC, and age were independent predictors for 28-day mortality in critically ill patients (*P* < 0.05). The AUC of ROC curve in H-FABP was 0.709 (sensitivity 72.9%, specificity 66.1%, and cut-off 4.35), which was slightly lower than AUC of ROC curve in LAC (AUC 0.750, sensitivity 58.3%, specificity 76.1%, and cut-off 1.95) and APACHE II (AUC 0.731, sensitivity 77.1%, specificity 58.7%, and cut-off 12.5). However, statistically, there was no difference in the diagnostic value of H-FABP compared with the other two indicators (*Z*_1_ = 0.669, *P* = 0.504; *Z*_2_ = 0.383, *P* = 0.702). But H-FABP (72.9%) has higher sensitivity than LAC (58.3%). The combined evaluation of H-FABP+APACHE II score (AUC 0.801, sensitivity 71.7%, and specificity 78.2%; *Z* = 2.612, *P* = 0.009) had better diagnostic value than H-FABP alone and had high sensitivity (71.7%) and specificity (78.2%).

**Conclusion:**

H-FABP, LAC, APACHE II, and age can be used as independent risk factors affecting the prognosis of critically ill patients. Compared with using the above indicators alone, the H-FABP+APACHE II has a high diagnostic value, and the early and rapid evaluation is particularly important for the adjustment of treatment plans and prognosis.

## 1. Introduction

Critical illness is common in emergency departments, with rapid disease progression and extremely high mortality. Under stress conditions such as severe infection, hypoxia-ischemia, and shock, a series of pathophysiological changes will occur in the heart, lung, brain, and kidney, which can easily develop into multiple organ dysfunction syndrome [1, 2]. Furthermore, an increasingly aging population has significantly increased the incidence of critical illness, with 58–65% of critically ill patients aged over 65 [3]. Factors such as increasing age, immune aging, and underlying organ failure lead to an increased risk of critical illness and sepsis, greater complications, and poor prognosis [4, 5]. Most critically ill patients are treated in the emergency department, and their condition is often relatively serious and progresses rapidly. Therefore, emergency physicians must perform an early diagnostic intervention, risk stratification, and prognosis assessment.

Traditional serological indicators such as procalcitonin (PCT), C-reactive protein (CRP), and C troponin (cTNI), as well as Sequential Organ Failure Assessment (SOFA) score and disease severity scores, help evaluate the severity and prognosis of critically ill patients [6–8]. Recently, we have shown that a new serological marker, heart-type fatty acid-binding protein (H-FABP), may have prognostic value in critically ill patients. Fatty acid-binding proteins were first identified in 1972 and are widely present in the body. Previous studies have shown that H-FABP was mostly used as an early marker of cardiovascular events such as myocardial injury because it is released in the early stage, reaching a peak value around 6 h [9–12]. In addition, H-FABP is also produced in extracardiac organs including the kidney, gut, brain, and skeletal muscle, and H-FABP may increase in critically ill patients with dysfunction of these organs. Therefore, the evaluation of H-FABP level for the prognosis and risk stratification of patients is worthy of further study. Therefore, our study explored the value of H-FABP and other markers in the prognosis of critically ill patients.

## 2. Materials and Methods

### 2.1. Study Design and Participants

Our study was conducted in the Affiliated Beijing Chaoyang Hospital of Capital Medical University from August 2021 to April 2022. During this period, 150 critically ill patients admitted to the emergency department were included in our study. The study was approved by Beijing Chaoyang Hospital ethics committee (2021-S-636). Written informed consent was obtained by patients or their family members, and patient confidentiality was strictly maintained.

The inclusion criteria were aged ≥18 and critically ill patients admitted to the emergency department. According to the diagnostic criteria recommended by the American College of Critical Care Medicine and the expert consensus on emergency preexamination and triage [13, 14], the inclusion criteria were as follows: (1) respiratory failure; (2) hemodynamic disorders, such as blood pressure <90/60 mmsHg; and (3) combined with other organ dysfunctions. The exclusion criteria were as follows: (1) length of the treatment to enter the emergency department < 12 h or transfer from other hospital; (2) patients had acute or chronic renal insufficiency in the past; (3) trauma patients; and (4) patients with previous malignant tumors, blood diseases, and connective tissue diseases.

### 2.2. Baseline Patient Demographics

General data were collected after admission, including gender, age, and type of disease (cardiovascular disease, respiratory disease, and other systemic diseases). All clinical events and outcomes, as well as nonsurvivor and survival at 28 days, were recorded.

### 2.3. Serum Markers

Blood samples were collected from patients within 1 h after entering the emergency department. The levels of cTNI, H-FABP, IL-6, and PCT were measured using a point-of-care testing analyzer (POCT, automatic fluorescence immunoassay analyzer, Vazyme Biotech Co., Ltd., China). Blood samples with severe hemolysis, lipid turbidity, and high concentrations of bilirubin were excluded from the analysis. LAC levels were determined by an automatic blood gas analyzer (ABL90 FLEX, Radiometer Medical Equipment Co., Ltd., Denmark). Other relevant laboratory indicators, including blood cell count and routine biochemistry, were measured in the clinical lab.

### 2.4. Clinical Score

The Sequential Organ Failure Assessment (SOFA) score and Acute Physiology and Chronic Health Evaluation II (APACHE II) were calculated based on the clinical data after admission to evaluate the severity of the patient's condition [14–16].

### 2.5. Statistical Analysis

Data were processed using the Statistical Package for the Social Sciences (SPSS) 23.0 (IBM SPSS Corp., Armonk, NY, USA) software. Quantitative data were expressed as mean ± standard deviation, and independent sample *t*-tests were used to compare the two groups. And, the median, 25%, and 75% place values were used for the data that did not conform to the normal distribution. The Mann-Whitney test was used for the calculation of nonnormally distributed data. Categorical variables were statistically analyzed using the chi-square test. The Pearson correlation was used to analyze the correlation between H-FABP and other indicators. Logistic regression was used to analyze the independent risk factors of nonsurvivors at 28 days, and the receiver operating characteristic (ROC) curve of different serum markers was calculated to determine the sensitivity, specificity, and area under the curve area. The cut-off value was determined using the Youden method. AUC comparisons were performed using *Z* test. The final multiple regression model included all potential variables significantly related to the results. G^∗^Power tool was used for power calculation. The difference was statistically significant at *P* < 0.05.

## 3. Results

### 3.1. Comparison of Patient Baseline Data

The data of 150 critically ill patients are listed in Table 1. According to Table 1, there was no significant difference in gender and underlying diseases (diabetes mellitus, hypertension, and coronary heart disease) between the two groups (*P* > 0.05). The *t*-test showed that the age (77.6 ± 12.2 vs. 68.6 ± 14.5) was significantly different between the nonsurvivor group and survivor group (*P* < 0.05). In terms of scores, the nonsurvivor group had higher average of APACHE II (18.7 ± 7.8 vs. 12.9 ± 5.7) and higher median value of SOFA (5.0 (3.8-7.3) vs. 2.0 (1.0-4.0)) than survivor group; the differences were statistically significant (*P* < 0.01). And the GCS of the nonsurvivor group was great lower than another group (13.5 (11.8-15.0) vs. 15.0 (15.0-15.0), *P* < 0.01). Among the serum indexes measured, the median values of LAC (2.4 (1.4, 3.8) vs. 1.2 (0.8, 2.0)), H-FABP (6.9 (3.9-32.0) vs. 3.6 (2.0-6.6)), IL-6 (51.6 (14.7-139.3) vs. 19.4 (5.3-64.7)), and D-dimer (1.9 (1.0, 11.8) vs. 0.9 (0.4-3.7)) were significantly different (*P* < 0.01). There were no statistical differences in other indicators. Of the 150 critically ill patients, the main diagnosis of the 68 (45.3%) patients were respiratory system diseases, 41 (27.3%) patients were cardiovascular system diseases, and 41 (27.3%) patients were urinary system diseases and other system diseases. There was no significant difference between two groups (*P* > 0.05).

### 3.2. Correlation between H-FABP and Other Indicators

Pearson's correlation analysis showed that H-FABP was moderately correlated with Scr and SOFA scores (*r*_1_ = 0.538, *r*_2_ = 0.456, *P* < 0.01). And there was a weak correlation between H-FABP and PCT, cTNI, and IL-6 (*r*_1_ = 0.249, *r*_2_ = 0.237, *r*_3_ = 0.191, *P* < 0.01), as displayed in Table 2.

### 3.3. The Relationship between H-FABP, LAC, APACHE II Score, Age, and 28-Day Prognosis of Critically Ill Patients

By including the positive indicators from the above univariate analysis into the multivariate regression analysis, we found that H-FABP (1.075 (1.033-1.119), *P* = 0.001), LAC (1.277 (1.009-1.615), *P* = 0.042), age (1.055 (1.016-1.097), *P* = 0.006), and APACHE II (1.125 (1.050-1.206), *P* = 0.001) were independent risk factors for 28-day mortality in critically ill patients, and the 28-day mortality showed an increasing trend with increasing values (Table 3).

**Figure 1:**
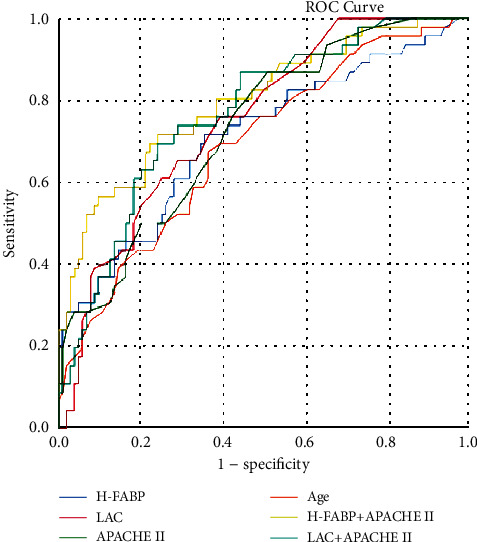
ROC curve of H-FABP, LAC, APACHE II score, and age predicting 28-day prognosis in critically ill patients.

In the diagnostic analysis of the four indicators screened by logistic regression, ROC curve analysis showed that the increase in age, LAC, H-FABP value, and APACHE II score could predict the 28-day prognosis of patients (Table 4 and Figure 1). The AUC of ROC curve in H-FABP was 0.709 (sensitivity 72.9%, specificity 66.1%, and cut-off 4.35), which was slightly lower than AUC of ROC curve in LAC (AUC 0.750, sensitivity 58.3%, specificity 76.1%, and cut-off 1.95) and APACHE II (AUC 0.731, sensitivity 77.1%, specificity 58.7%, and cut-off 12.5). Statistically, there was no difference in the diagnostic value of H-FABP compared with the other two indicators (*Z*_1_ = 0.669, *P* = 0.504; *Z*_2_ = 0.383, *P* = 0.702). But H-FABP (72.9%) has higher sensitivity than LAC (58.3%). And we found that combined evaluation of H-FABP+APACHE II score (AUC 0.801, sensitivity 71.7%, and specificity 78.2%; *Z* = 2.612, *P* = 0.009) had better diagnostic value than H-FABP alone and had high sensitivity (71.7%) and specificity (78.2%).

## 4. Discussion

The emergency department is responsible for treating most acute and critically ill patients [17, 18]. Especially with an increasingly aging population, elderly patients often need to be treated for multiple diseases of the respiratory, cardiovascular and cerebrovascular, and digestive systems. Therefore, emergency physicians must timely diagnose and treat [19]. In our study, POCT serological levels and severity scores were evaluated in 150 critically ill patients after admission, which is helpful for timely risk stratification, diagnosis, and treatment intervention.

H-FABP is a small molecular intracellular protein with a molecular weight of about 12-16 kd, which has been used as an early biomarker of myocardial infarction in recent years. Because it is rapidly increased within 3 h after myocardial ischemic injury [9], studies have reported that H-FABP is a promising biomarker for acute coronary syndrome (ACS) and cardiac dysfunction [20]. Some scholars believe that H-FABP levels are higher in patients with sepsis-related myocardial dysfunction, which is superior to traditional biomarkers in terms of diagnostic value [21]. Chen and Li evaluated 295 infected patients with confirmed sepsis, and H-FABP was helpful for prognosis and risk stratification of septic patients [22]. There was a relatively weak correlation between H-FABP and cTNI, according to Pearson's correlation analysis (*r* = 0.237, *P* < 0.01, Table 2). Meanwhile, we found through logistic regression analysis that cTNI was not statistically significant in predicting 28-day mortality of critically ill patients.

For critical illness in the emergency department, the patient's condition was critical and changed rapidly, including respiratory failure, disturbance of consciousness, and hemodynamic instability, and needed to enter the emergency room for vital monitoring and support [19, 23]. A series of physiological changes occur in such patients under stress, including myocardial injury, intestinal barrier function damage, and inflammatory factor release, which cause multiple organ failure [24]. Previous studies have shown that H-FABP is expressed in all the above-mentioned organs [22]. In our study, we founded that the value of H-FABP in the nonsurvivor group was higher than the survivor group (6.9 (3.9-33.0) vs. 3.6 (2.0-6.6), *P* = 0.001) and H-FABP was an independent risk factors for 28-day mortality of critically ill patients by logistic analysis (OR 1.075 (1.033-1.119), *P* = 0.001). In previous studies, LAC has been shown to be an effective predictor for evaluating the prognosis of critically ill patients, so it is often used in clinical practice [2, 25]. Baumann et al. evaluated lactate values in 3743 patients admitted to the hospital from the ED with infectious disease-related illnesses that lactate 2 provides substantially improved sensitivity for the screening of critical illness [2]. In our analysis, however, no statistically significant differences were found in the AUC values for H-FABP and LAC. Interestingly, when the cut-off value of H-FABP was 4.35 ng/ml, the sensitivity of prognosis was 72.9%, which was significantly higher than 58.3% for LAC. And the optimal cut-off value is 4.35, which means that patients with H-FABP above this value have more severe condition and a worse prognosis. Both H-FABP and LAC are independent risk factors for the prognosis of critically ill patients, but combined with their AUC, sensitivity, and specificity, the diagnostic value of H-FABP is similar to LAC, or even better in some aspects.

In addition to serological markers, the critically ill patients admitted to the emergency department were evaluated by APACHE II, and the patient's condition and prognosis were assessed through multiple systems such as acute physiology score and chronic health score [7].

Previous studies have shown that APACHE II score may supply the prognostic utility for the mortality of sepsis [26, 27]. In our study, APACHE II also has certain clinical significance in predicting the 28-day mortality of critically ill patients through early and rapid assessment. We combined the serological indicators H-FABP and LAC and the scoring index APACHEII and found that H-FABP+ APACHEII and LAC+APACHE II had high diagnostic value, but there was no significant difference (AUC_1_ = 0.801, sensitivity 71.7%, and specificity 78.2%; AUC_2_ = 0.768, sensitivity 73.9%, and specificity 71.8%; *Z* = 0.885, *P* = 0.376). At the same time, compared with using traditional scoring systems such as APACHE II alone, the combined detection had better area under the curve and specificity, and the difference was statistically significant (*Z* = 2.448, *P* = 0.014). With increasing age, the immune function also decreases, reducing the ability of the body to fight infection. Meanwhile, the dysfunction of various systems and organs causes congestive heart failure, chronic obstructive pulmonary disease, diabetes, and liver and kidney failure, and other critical diseases also appear [5, 28]. An optimal cut-off value of 73.5 years was obtained by ROC curve, indicating a poor prognosis for critically ill patients above this age. In summary, the serological index H-FABP has certain advantages over other indicators in the overall diagnostic efficacy, and the combination of H-FABP and APACHE II score can be quickly evaluated after admission, and clinicians can carry out early diagnosis and treatment of patients. Therefore, we believe that the H-FABP can better assess the severity of the condition and the prognosis of death in critically ill patients. A post hoc power analysis is based on our data that a sample size of 104 patients in the survivor group and 46 patients in the nonsurvivor group would yield a statistical efficacy greater than 90% at a bilateral *α* value of 0.05. The results of our study had certain clinical value.

Nonetheless, our study has certain limitations. First, it was a single-center study, with patients limited to a certain area and a small sample size, so large-scale, multicenter studies are needed to verify the results. Second, we did not dynamically observe the relationship between the changes of H-FABP and patient prognosis, which also requires our further study. Third, initial serological indicators only reflect the inflammatory response and disease severity at the onset of the disease. It is helpful for clinicians to assess the patient's condition but cannot fully represent the final prognosis of the patients.

## 5. Conclusion

In summary, H-FABP, LAC, APACHE II, and age can be used as independent risk factors affecting the prognosis of critically ill patients. Compared with using the above indicators alone, the H-FABP+APACHE II has a high diagnostic value, and the early and rapid evaluation is particularly important for the adjustment of treatment plans and prognosis.

## Figures and Tables

**Table 1:** Comparison of patient baseline data.

	Nonsurvivor group (*n* = 46)	Survivor group (*n* = 104)	*P*
Gender (male/female)	33/13	62/42	0.107
Age (years)	77.6 ± 12.2	68.6 ± 14.5	0.001
Underlying disease			
Diabetes (yes/no)	17/29	42/62	0.721
Hypertension (yes/no)	14/32	31/73	0.938
Coronary heart disease (yes/no)	16/30	28/76	0.338
Main diagnosis			0.577
Respiratory system	23 (34.0%)	45 (66.0%)	
Cardiovascular system diseases	10 (24.4%)	31 (75.6%)	
Other	13 (31.7%)	28 (68.3%)	
APACHE II	18.7 ± 7.8	12.9 ± 5.7	0.001
SOFA	5.0 (3.8, 7.3)	2.0 (1.0, 4.0)	0.001
GCS	13.5 (11.8, 15.0)	15.0 (15.0, 15.0)	0.001
WBC (10^9^/l)	12.2 ± 6.3	11.2 ± 9.7	0.491
CRP (mg/l)	23.9 ± 50.9	21.1 ± 39.8	0.717
HB (g/l)	108.6 ± 25.6	108.1 ± 28.8	0.925
PLT (10^9^/l)	203.7 ± 111.6	210.9 ± 80.9	0.656
Scr (umol/l)	99.7 (68.6, 224.2)	74.7 (55.9, 94.8)	0.001
LAC (mmol/l)	2.3 (1.4, 3.4)	1.2 (0.8, 2.0)	0.001
PCT (ng/ml)	0.14 (0.05, 1.33)	0.08 (0.02, 0.37)	0.051
H-FABP (ng/ml)	6.9 (3.9, 33.0)	3.6 (2.0, 6.6)	0.001
cTNI (ng/ml)	0.06 (0.04, 0.59)	0.05 (0.03, 0.27)	0.139
IL-6 (pg/ml)	51.6 (14.7, 139.3)	19.4 (5.3, 64.7)	0.001
D-dimer (mg/l)	1.9 (1.00, 11.8)	0.9 (0.4, 3.7)	0.001

^a^APACHE II: acute physiology and chronic health evaluation II; SOFA: sequential organ failure assessment score; GCS: Glasgow coma scale; H-FABP: heart-type fatty acid-binding protein; PCT: procalcitonin; cTNI: cardiac troponin I; IL-6: interleukin 6; LAC: lactate; Scr: serum creatinine; CRP: C-reactive protein; HB: hemoglobin. The differences were statistically significant when *P* < 0.05.

**Table 2:** Correlation analysis between H-FABP and other indicators.

	Scr	PCT	cTNI	LAC	APHACHE II	SOFA	GCS	D-dimer	IL-6
Pearson's correlation	0.538	0.249	0.237	0.156	0.091	0.456	-0.207	0.061	0.191
*P*	0.01	0.01	0.004	0.06	0.27	0.01	0.09	0.45	0.02

^a^APACHE II: acute physiology and chronic health evaluation II; LAC: lactate; Scr: serum creatinine; SOFA: sequential organ failure assessment score; PCT: procalcitonin; cTNI: cardiac troponin I; GCS: Glasgow coma scale; IL-6: interleukin 6. The differences were statistically significant when *P* < 0.05.

**Table 3:** Multivariate regression analysis of H-FABP, LAC, APACHE II scores, age, and 28-day prognosis in critically ill patients.

	*β*	SE	Wald	*P*	OR (95% CI)
H-FABP	0.0072	0.020	12.855	0.001	1.075 (1.033-1.119)
LAC	0.244	0.120	4.153	0.042	1.277 (1.009-1.615)
APACHE II	0.118	0.035	11.103	0.001	1.125 (1.050-1.206)
Age	0.054	0.020	7.547	0.006	1.055 (1.016-1.097)

^a^APACHE II: acute physiology and chronic health evaluation II; H-FABP: heart-type fatty acid-binding protein; LAC: lactate; SE: standard error. The differences were statistically significant when *P* < 0.05.

**Table 4:** Predictive value of H-FABP, LAC, APACHE II score, age, and 28-day prognosis in critically ill patients.

	AUC	95% CI	*P*	Cut-off	Sensitivity (%)	Specificity (%)
H-FABP	0.709	0.615-0.802	0.001	4.35	72.9	66.1
LAC	0.750	0.670-0.830	0.001	1.95	58.3	76.1
APACHE II	0.731	0.647-0.814	0.001	12.5	77.1	58.7
Age	0.686	0.589~0.782	0.001	73.5	67.4	63.5
H-FABP+APACHE II	0.801	0.719-0.877	0.001		71.7	78.2
LAC+APACHE II	0.768	0.689-0.847	0.001		73.9	71.8

^a^APACHE II: acute physiology and chronic health evaluation II; H-FABP: heart-type fatty acid-binding protein; LAC: lactate; AUC: area under ROC curve; Cut-off: the cut-off value was determined using the Youden method. The differences were statistically significant when *P* < 0.05.

## Data Availability

The datasets generated and/or analyzed during the current study are available from the corresponding authors on reasonable request.
